# Ferristatin II Promotes Degradation of Transferrin Receptor-1 *In Vitro* and *In Vivo*

**DOI:** 10.1371/journal.pone.0070199

**Published:** 2013-07-23

**Authors:** Shaina L. Byrne, Peter D. Buckett, Jonghan Kim, Flora Luo, Jack Sanford, Juxing Chen, Caroline Enns, Marianne Wessling-Resnick

**Affiliations:** 1 Department of Genetics and Complex Diseases, Harvard School of Public Health, Boston, Massachusetts, United States of America; 2 Department of Cell Biology, Oregon Health Sciences Center, Portland, Oregon, United States of America; Tohoku University, Japan

## Abstract

Previous studies have shown that the small molecule iron transport inhibitor ferristatin (NSC30611) acts by down-regulating transferrin receptor-1 (TfR1) via receptor degradation. In this investigation, we show that another small molecule, ferristatin II (NSC8679), acts in a similar manner to degrade the receptor through a nystatin-sensitive lipid raft pathway. Structural domains of the receptor necessary for interactions with the clathrin pathway do not appear to be necessary for ferristatin II induced degradation of TfR1. While TfR1 constitutively traffics through clathrin-mediated endocytosis, with or without ligand, the presence of Tf blocked ferristatin II induced degradation of TfR1. This effect of Tf was lost in a ligand binding receptor mutant G647A TfR1, suggesting that Tf binding to its receptor interferes with the drug’s activity. Rats treated with ferristatin II have lower TfR1 in liver. These effects are associated with reduced intestinal ^59^Fe uptake, lower serum iron and transferrin saturation, but no change in liver non-heme iron stores. The observed hypoferremia promoted by degradation of TfR1 by ferristatin II appears to be due to induced hepcidin gene expression.

## Introduction

Iron is involved in various processes of cellular homeostasis including DNA synthesis and repair, ATP synthesis, and oxygen transport [1]. It is essential for life and depletion of iron restricts growth of cells [2], [3], [4]. Iron is absorbed from the diet by duodenal enterocytes and is transported to the periphery bound to transferrin (Tf). At neutral pH, holo-Tf binds to receptors on the cell surface [5]. This complex undergoes clathrin-dependent endocytosis and is delivered to early endosomes [6]. Within the low pH environment of the endosome, iron is released from Tf. Iron is reduced from ferric iron (Fe^3+^) to ferrous iron (Fe^2+^) by the ferrireductase Steap3 [7], [8]. Ferrous iron is then transported into the cytosol via divalent metal transporter-1 (DMT-1) [9], ZIP14 [10] and/or TRPML [11]. The ligand-receptor complex, devoid of iron, is recycled back to the plasma membrane where apo-Tf dissociates and continues the cycle of iron acquisition and delivery to peripheral tissues [12], [13].

There are two known Tf receptors, TfR1 and TfR2. At the cellular level, TfR1 is ubiquitously expressed and is largely responsible for Tf-mediated delivery of iron to peripheral tissues [14]. TfR1 is a constitutively recycling receptor that undergoes clathrin-mediated endocytosis with or without its ligand [12], [15]. It is widely held that the presence of TfR1 identifies the endocytic, sorting and recycling compartments of most cells. Often marked by fluorescently labeled Tf or receptor immunoreactivity, TfR1 is frequently used as a reference marker for these domains [16], [17]. The interactions of TfR1 with the clathrin machinery also provide a paradigm for how membrane proteins engage with coated pits and become internalized by coated vesicles [18]. In particular, receptor interactions with the clathrin adaptor protein AP-2 have been studied at the molecular and structural level [19], [20], [21]. At the plasma membrane, interactions with the AP-2 adaptor complex mediate assembly of clathrin triskelions that form a budding coated pit that is pinched off by dynamin to generate a coated vesicle [22], [23].

At the systemic level, regulation of iron homeostasis has been elucidated through studies of hereditary hemochromatosis [24]. Mutations in HFE [25], transferrin receptor-2 [26], ferroportin [27], hepcidin [28], or hemojuvelin [29] have been identified in different forms of human disease. Iron metabolism is regulated through a complex network of protein-protein interactions between these factors. Although TfR1 is ubiquitously expressed and plays a key role in iron delivery through receptor-mediated endocytosis of diferric transferrin, HFE and TfR2 play a more specialized role in the liver where they have been shown to act as upstream regulators of hepcidin synthesis [30]. A model has been proposed wherein HFE interactions with TfR1 limit its association with TfR2 [31]. When iron levels increase, saturation of Tf promotes receptor binding, which in turn competitively displaces HFE from TfR1 to allow its interaction with TfR2 [32]. Over-expression of HFE in the livers of mice increases hepcidin expression, supporting the notion that levels of endogenous protein are limiting [30], [33].

Hepcidin, a 25 amino acid peptide hormone, is secreted primarily by the liver and regulates systemic iron status [34]. The peptide binds to the iron exporter ferroportin (Slc40a1), inducing its internalization and lysosomal degradation [35]. This mechanism controls intestinal iron efflux and recycling of iron from macrophages [36], [37]. Concordantly, hepcidin synthesis increases with iron loading and decreases with iron deficiency [38], [39]. Hepcidin levels are inappropriately low in hemochromatosis patients with mutations in HFE [40] and TfR2 [41].

Genetic approaches to understanding iron homeostasis have been complemented by the recent development of pharmacological tools to either inhibit or activate transport and regulatory factors involved in iron metabolism [42]. One approach to further our understanding of the regulation of iron transport and homeostasis at the molecular level is through the use of small molecule inhibitors. Using a cell-based fluorescence screening strategy, ferristatin (NSC306711) was initially identified as an inhibitor of Tf-mediated iron delivery [43]. Characterization of ferristatin’s action revealed that it promoted the degradation of TfR1 in a nystatin-sensitive fashion. Internalization and receptor down-regulation of TfR1 in the presence of ferristatin occurred in a clathrin- and dynamin-independent manner [44]. These results were unexpected given the established role of clathrin-mediated endocytosis in TfR1 membrane trafficking, but were specific to this receptor since ferristatin did not alter LDL receptor trafficking [44]. More recently, NSC8679 (referred to here as ferristatin II) was identified as a polysulphonated dye not only structurally related to ferristatin but with functional similarities as well [45]. This investigation was undertaken to characterize ferristatin II action and the nature of the nystatin-sensitive clathrin-independent mechanism for TfR1 down-regulation. The alternative lipid raft mediated trafficking and degradation pathway revealed by the ferristatins may hold clinical potential to limit iron acquisition. This idea is supported by results of *in vivo* experiments that reveal a systemic effect on iron metabolism upon treatment with ferristatin II.

## Materials and Methods

### Ethics Statement

This study was performed in strict accordance with the recommendations in the Guide for the Care and Use of Laboratory Animals of the National Institutes of Health. The protocol was approved by the Harvard Medical Area Animal Care and Use Committee (Animal Experimentation Protocol AEP #04692).

### Cell Culture

HeLa cells were grown in Dulbecco’s minimal essential medium (DMEM) containing 50 U/mL penicillin, 50 µg/mL streptomycin, L-glutamine, and 10% fetal bovine serum (FBS, Sigma). TRVb cells [46] (a kind gift of Dr. Timothy E. McGraw, Weill Medical College, Cornell University) were grown in Ham’s F-12 medium containing 10% FBS. Stably transfected TRVb cells containing either WT TfR1, Y20C/F23A TfR1 or Δ3–28 TfR1 were grown in Ham’s F-12 containing 5 g/L glucose, 400 µg/mL G418 and 5% FBS. Hep3B cells stably expressing human TfR2 [47] were maintained in minimal essential medium (MEM) containing 1 mM sodium pyruvate, 0.1 mM non-essential amino acids, 10% FBS and 400 µg/mL G418.

### Ferristatin II Treatment: *In vitro*

Ferristatin II (NSC8679) was obtained from Sigma (Product No. C1144, also called Chlorazol Black or Direct Black 38). For treatment with ferristatin II, cells were first washed three times with phosphate-buffered saline containing 1 mM MgCl_2_ and 0.1 mM CaCl_2_ (PBS^++^) and then washed once with serum-free medium. Fifty µM ferristatin II or DMSO control was added to cells in serum free medium. To inhibit lysosomal degradation of TfR1, cells were treated overnight with 10 nM Bafilomycin A_1_ (Sigma, B1793). To disrupt lipid rafts, cells were pretreated for 20 minutes with 25 µg/mL nystatin (Sigma) before addition of ferristatin II. Cells were incubated at 37°C with 5% CO_2_ for 4 hours unless otherwise stated.

### Ferristatin II Treatment: *In vivo*

Male Sprague-Dawley rats (3-wk-old) were injected twice daily for 1 or 3 days with ferristatin II (up to maximum of 40 mg/kg) or saline as a vehicle control. On day 2 or day 4, rats were injected once and fasted for 6 hours. At the start of the fasting period, rats were housed in metabolic cages for 6 h to collect urine. For ^59^Fe tracer studies, on day 4 rats were fasted for 4 h prior to administration of ^59^Fe by gavage (1 µl/g body weight of a solution of 30 µCi/ml, diluted in 20 mM Tris, 150 mM NaCl, pH 5.7 with 10 mM freshly dissolved ascorbate). Blood samples were taken at intervals from 15 min to 1 h and radioactivity was determined by gamma counting. At the end of the study, rats were euthanized by isoflurane overdose followed by exsanguination for collection of tissues to analyze iron status.

### Western Blot Analysis

After incubation with ferristatin II as described above, cells were washed three times with ice cold PBS^++^ and lysed in NET lysis buffer (150 mM NaCl, 5 mM EDTA, 10 mM Tris pH 7.4 and 1% Triton X-100) containing protease inhibitors (Protease Inhibitor Set lll, Calbiochem) for 20 minutes on ice. Lysates were cleared at 16,000×g for 10 minutes at 4°C and 20–60 µg of the supernatant were loaded on 8% SDS-polyacrylamide gels. Livers from rats treated with ferristatin ll or vehicle control were homogenized in RIPA buffer (10 mM Tris, pH 7.4, 150 mM NaCl, 1.0 mM EDTA, 0.1% SDS, 1.0% Triton X-100, 1.0% sodium deoxycholate) containing protease inhibitors (Halt, Thermo Scientific) and 100 µg samples were electrophoresed on 10% SDS-polyacrylamide gels. After electrophoresis, samples were transferred to nitrocellulose or PVDF membrane, blocked with 5% non-fat milk and immunoblotted using monoclonal mouse anti-TfR1 antibody H68.4 (1∶500 or 1∶1000, Invitrogen), sheep anti-TfR1 antibody (1∶5000, [48]) monoclonal mouse anti-TfR2 antibody 9F81C11 (1∶1000, Santa Cruz Biotechnology) or mouse anti-Flag (1∶1000, Sigma). As a loading control, blots were probed with mouse anti-actin C4 clone (1∶10,000, MP Biomedicals) or mouse anti-tubulin (1∶10,000, Sigma). Secondary antibody, IRDye800 or IRDye680 conjugated donkey anti-mouse, donkey anti-rabbit or donkey anti-sheep (1∶10,000, LI-COR) was used to detect immunoreactivity using an Odyssey Infrared Imaging System (LI-COR). Relative intensities of protein bands were normalized to actin using Odyssey version 2.1 software.

### G647A TfR1 Mutagenesis and Transfection Experiments

The pcDNA3/G647A TfR1 plasmid was generated using QuikChange XL site-directed mutagenesis (Strategene, La Jolla, CA) using a template of pcDNA3/TfR1 and primers of 5'- CTG TAT TCT GCT CGT GCA GAC TTC TTC CGT GC -3' and 5'- GCA CGG AAG AAG TCT GCA CGA GCA GAA TAC AG -3' following manufacturer’s instructions. TRVb cells were transiently transfected to express either wild type TfR1 (2 µg plasmid DNA) or the G647A TfR1 point mutant (2 µg plasmid DNA) using LipofectAMINE2000 (Invitrogen) according to manufacturer’s instructions. Cells were split 24 hours post-transfection into 6-well plates and treated with ferristatin II the following day. HeLa cells were transfected with HFE containing a Flag tag (0.5 µg plasmid DNA) or pcDNA vector control (0.5 µg plasmid DNA) as described above.

### ^125^I-Transferrin Surface Binding

TRVb cells were grown to 90% confluency in Ham’s F-12 medium containing 10% FBS. Cells were transfected as described above with WT TfR1 or G647A TfR1. After 24 hours, cells were plated into 6 well dishes and incubated for an additional 24 hours. Cells were chilled on ice for 15 min and washed twice in ice-cold serum-free Ham’s F-12 medium. Cell monolayers were incubated on ice in serum-free Ham’s F-12 medium containing 20 mM Hepes, pH 7.4, 2 mg/ml ovalbumin, and 500 nM ^125^I-Tf with or without 5 µM unlabeled Tf to displace non-specifically bound ligand. After incubation on ice for 2 hours, cells were washed 4 times with PBS^++^ and lysed with solubilization buffer (0.1% Triton X-100, 0.1% NaOH). Cell-associated radioactivity was measured by gamma counting. Data were adjusted to protein levels determined by Bradford assay.

### Fluorescence Microscopy

TRVb cells transfected with WT TfR1 or G647A TfR1 were plated onto poly-L-Lysine coated cover slips 24 hours post transfection and incubated for an additional 24 hours at 37°C, 5% CO_2_. Cells were washed with PBS^++^ and fixed with 4% paraformaldehyde. Cells were incubated with PBS (non-permeabilized) or PBS +0.1% Triton X-100 (permeabilized), washed with 1% NH_4_Cl and blocked with 5% goat serum. The cells were then immunoreacted with mouse anti-human transferrin receptor, OKT9 (eBioscience), followed by goat anti-mouse Alexa 488 (Invitrogen). Cells were imaged using a Zeiss Observer Z1 Axioscope microscope.

### RNA Isolation, cDNA Synthesis and Quantitative PCR

Total RNA was isolated using TRIZOL reagent (Invitrogen) according to the manufacturer’s instructions. An additional step with Phenol/Chloroform/8-Quinolinol was used to further purify the RNA. Five µg RNA were reverse transcribed using SuperScript lll First-Strand Synthesis System (Invitrogen) using random hexamers and oligo(dt)_20_ primers. Gene expression was analyzed by quantitative real-time PCR using Power SYBR Green PCR Master Mix (Applied Biosystems) on an Applied Biosystems 7300 Real Time PCR System. The cDNA was diluted 1∶40 and 6 µl was used as template in a 15 µl reaction volume. The conditions used were: 40 cycles 95°C for 15 s, 55°C for 30 s, and 72°C for 30 s. Analysis was performed in triplicate for each sample. A dissociation curve analysis was performed to detect non-specific products. 36B4 was used as a reference gene. All primers were used at a final concentration of 200 nM. The primers used were: hepcidin 5'-TGACAGTGCGCTGCTGATG-3' (forward), 5'-GGAATTCTTACAGCATTTACAGCAGA-3' (reverse); 36B4 5'-AGATGCAGCAGATCCGCAT-3' (forward) and 5'-GTTCTTGCCCATCAGCACC-3' (reverse). Calculations for relative quantification were done using the comparative C_T_ method (ΔΔC_T_).

### Urinary Monoacetylbenzidine Determination

Rat urine was centrifuged, reduced with ascorbic acid at 1 mg/ml, acidified to pH ≤3.0 using concentrated HCl and bound to a Strata-X-C SPE column (Phenomenex). After washing, the sample was eluted with 5% ammonium hydroxide in methanol, concentrated in a speedvac and analyzed using HPLC with a Zorbax Eclipse Plus Phenyl-Hexyl column (Agilent Technologies). The mobile phase was a step gradient starting with 95% 10 mM ammonium acetate, pH 4.7 and 5% acetonitrile; reaching 35% ammonium acetate, 65% acetonitrile after 15.5 mins. A flow rate of 1 ml/min and a detection wavelength of 290 nm were used. Fractions corresponding to the monoacetylbenzidine (MAB) elution time were collected from a standard, control and ferristatin ll treated urine samples and further analyzed by LCMS (The Small Molecule Mass Spectrometry Facility at the FAS Center for Systems Biology, Harvard University), confirming the peak corresponded to the molecular mass of MAB.

### Other Assays

Urinary creatinine was determined using the Creatinine Reagent Set (Pointe Scientific Inc.) using a modification of the manufacturer’s instructions. The working reagent (100 µl) was added to urine samples or a serial dilution of a creatinine standard (5 µl). After incubation at room temperature for 20 mins, the OD was read at 510 nm. These values were used to normalize urinary MAB levels. Serum alanine aminotransferase (ALT) and aspartate aminotransferase (AST) levels were determined using Infinity ALT and AST reagents (Thermo Scientific) according to the manufacturer’s instructions. Assays for hematocrit and liver non-heme iron concentrations were performed as previously described [49]. Serum iron levels were determined as described [50].

## Results

### Ferristatin II Induces Degradation of TfR1

**Figure 1:**
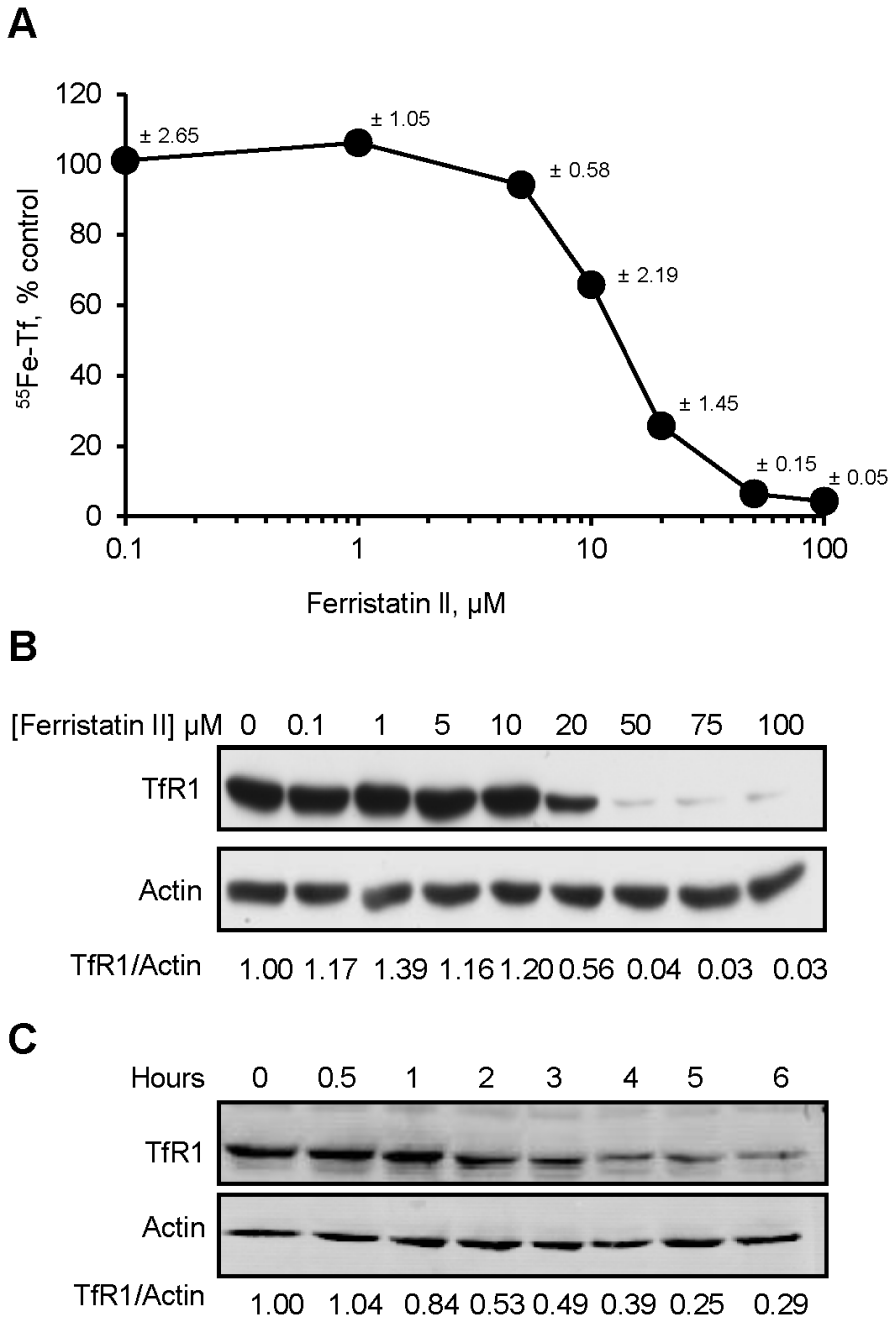
Ferristatin II induces degradation of TfR1 *in vitro*. Panel A: HeLa cells were treated for 4 hours with indicated concentrations of ferristatin II in the presence of 40 nM ^55^Fe-Tf. Shown are means ± SEM for triplicate values. Panel B: HeLa cell lysates were collected for Western blotting to determine Tf receptor levels. Panel C: Time course studies were carried out with cells treated with 50 µM ferristatin II for up to 6 hours.

Previous screening of the National Cancer Institute’s small molecule Diversity Set library identified ferristatin (NSC30611) as an inhibitor of Tf-mediated iron uptake [43]. Ferristatin’s mechanism of action includes down-regulation of TfR1 by a lipid raft pathway that promotes receptor degradation [44]. Subsequent analysis of structural orthologs determined similar properties were associated with a second iron transport inhibitor NSC8679, disodium 4-amino-3-((4′-((2,4-diaminophenyl)azo)-4-biphenylyl)azo-5-hydroxy-6-(phenylazo)-2,7-naphthalene disulfonate [45]. In this report, we refer to the second compound as ferristatin II. The influence of ferristatin II on Tf-mediated iron uptake was characterized *in vitro* using HeLa cells. Dose-dependent inhibition of cellular ^55^Fe uptake was observed when cells were treated with up to 100 µM ferristatin II for 4 hours at 37°C in the presence of ^55^Fe-Tf (Figure 1A). The IC_50_ value of ∼ 12 µM was similar to previous results obtained for ferristatin [44]. Western blot analysis confirmed that under these conditions, TfR1 was degraded (Figure 1B). The dose-dependent loss of TfR1 correlates with the reduction in iron uptake activity over this concentration range and provides a molecular explanation for lower cellular ^55^Fe uptake from Tf due to reduced receptor levels. Degradation of TfR1 was time-dependent with approximately 60–70% of receptors lost within 4 hours of treatment with 50 µM ferristatin II (Figure 1C).

### Ferristatin II does not Degrade TfR2 or HFE

**Figure 2:**
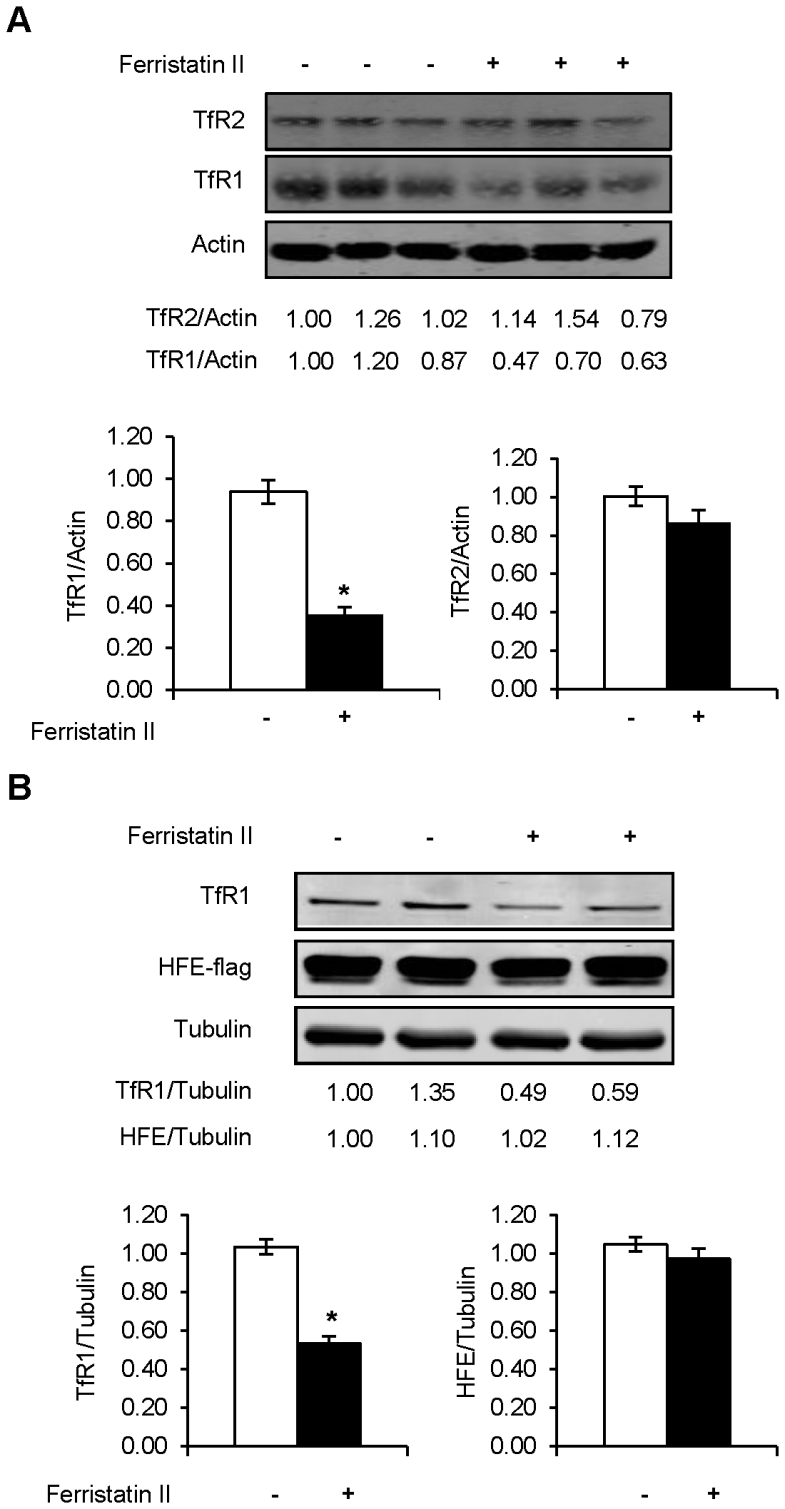
Ferristatin II does not degrade TfR2 or HFE. Panel A: Lysates from Hep3B cells stably expressing TfR2 were collected for Western blotting after 4 hours of 50 µM ferristatin II treatment. Ratios of band density for TfR1/Actin or TfR2/Actin are normalized to vehicle control (DMSO) in the absence of ferristatin II. The bar graph summarizes data from four separate experiments (n = 11 TfR1/Actin p<0.001, determined by two-tailed Student’s t test, n = 12 TfR2/Actin). Panel B: HeLa cells were transfected to express HFE-flag as described in Materials and Method. Cells were then incubated 4 h with or without 50 µM ferristatin II. Tubulin is shown as a loading control and the indicated values were normalized to control lanes in the absence of ferristatin II. The bar graph represents multiple transfections (n = 8, p<0.001 for TfR1/Actin determined by two-tailed Student’s t test).

TfR1 is known to associate with HFE at the plasma membrane and during endocytosis [51], [52]. It is thought that Tf binding to TfR1 promotes dissociation of HFE from TfR1 and promotes its interactions with TfR2, a close homolog of TfR1 [53]. To investigate the specificity of ferristatin II, Hep3B cells stably expressing TfR2 were treated with ferristatin II for 4 hours. Although endogenous TfR1 was degraded, levels of TfR2 were not decreased. (Figure 2A). It has been previously shown that exogenous expression of epitope-tagged HFE can be used to monitor its interaction with TfR1 [51], [52]. Therefore HeLa cells were transfected to transiently express flag-tagged HFE, then treated with ferristatin II. Levels of flag-tagged HFE were unaffected by ferristatin II while TfR1 was still degraded (Figure 2B). These results show that ferristatin II selectively induces the loss of TfR1.

### Ferristatin II Induced Degradation of TfR1 is Sensitive to Bafilomycin and Nystatin

**Figure 3:**
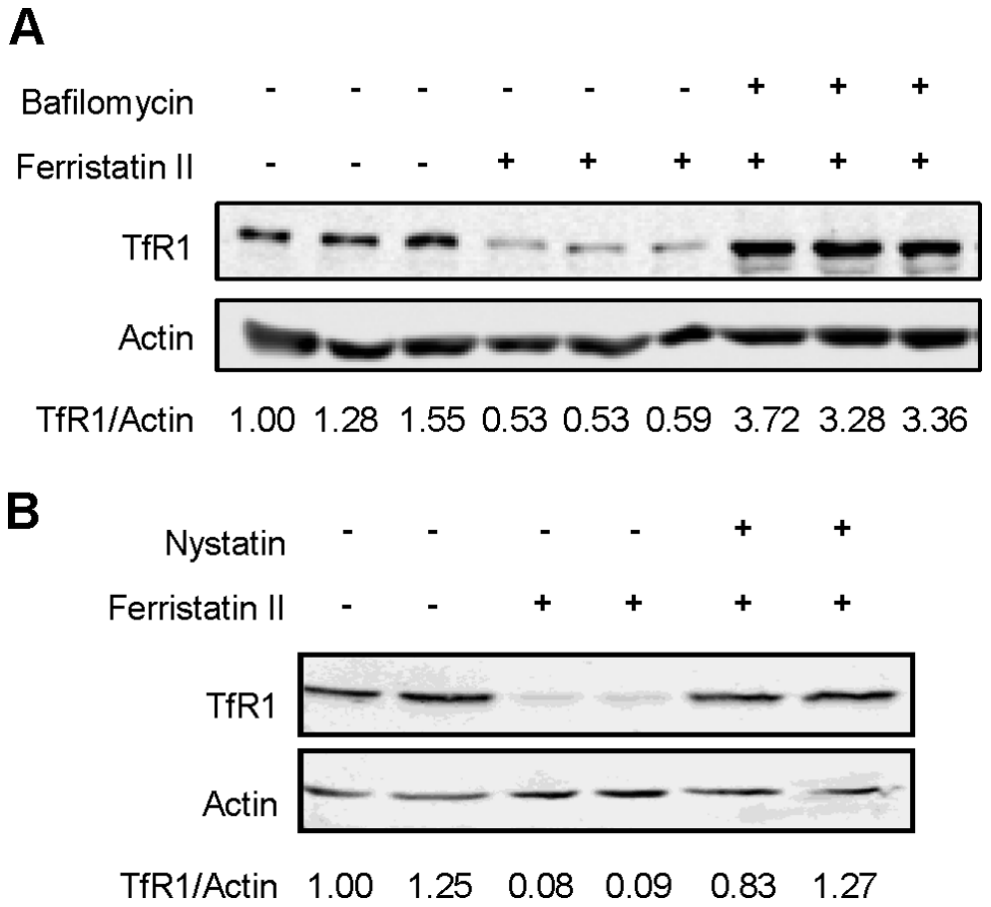
Ferristatin II induced degradation is bafilomycin and nystatin sensitive. Panel A: HeLa cells were treated overnight with 10 nM bafilomycin A_1_ prior to 4 h treatment with or without 50 µM ferristatin II in the presence of 10 nM bafilomycin A_1_. Blot is representative of several experiments. Panel B: HeLa cells were pre-treated for 30 minutes with 25 µg/mL nystatin or left untreated. After incubation, cells were treated with 50 µM ferristatin II for 4 hours. Shown below are the density ratios for TfR1/Actin normalized to control lanes (DMSO treated) in the absence of ferristatin II. Shown is a representative blot from several similar experiments.

It has been previously shown that ferristatin-induced TfR1 degradation is blocked by lysosomal inhibitors [44]. In a similar fashion, bafilomycin A_1_, which raises the pH in intracellular compartments, also blocks TfR1 degradation induced by ferristatin (Figure 3A). These findings are consistent with the idea that receptors are internalized for degradation. Past studies with ferristatin also have shown that endocytosis through lipid raft domains was necessary for TfR1 degradation [44]. To explore the possible role of lipid rafts in ferristatin II’s mechanism of action, we used the cholesterol binding agent nystatin to disrupt lipid rafts [54], [55]. The effects of ferristatin II on TfR1 degradation were antagonized by the presence of nystatin (Figure 3B).

### TfR1 Domains Necessary for Clathrin Pathway Interactions are not Necessary for Ferristatin II Induced Degradation

**Figure 4:**
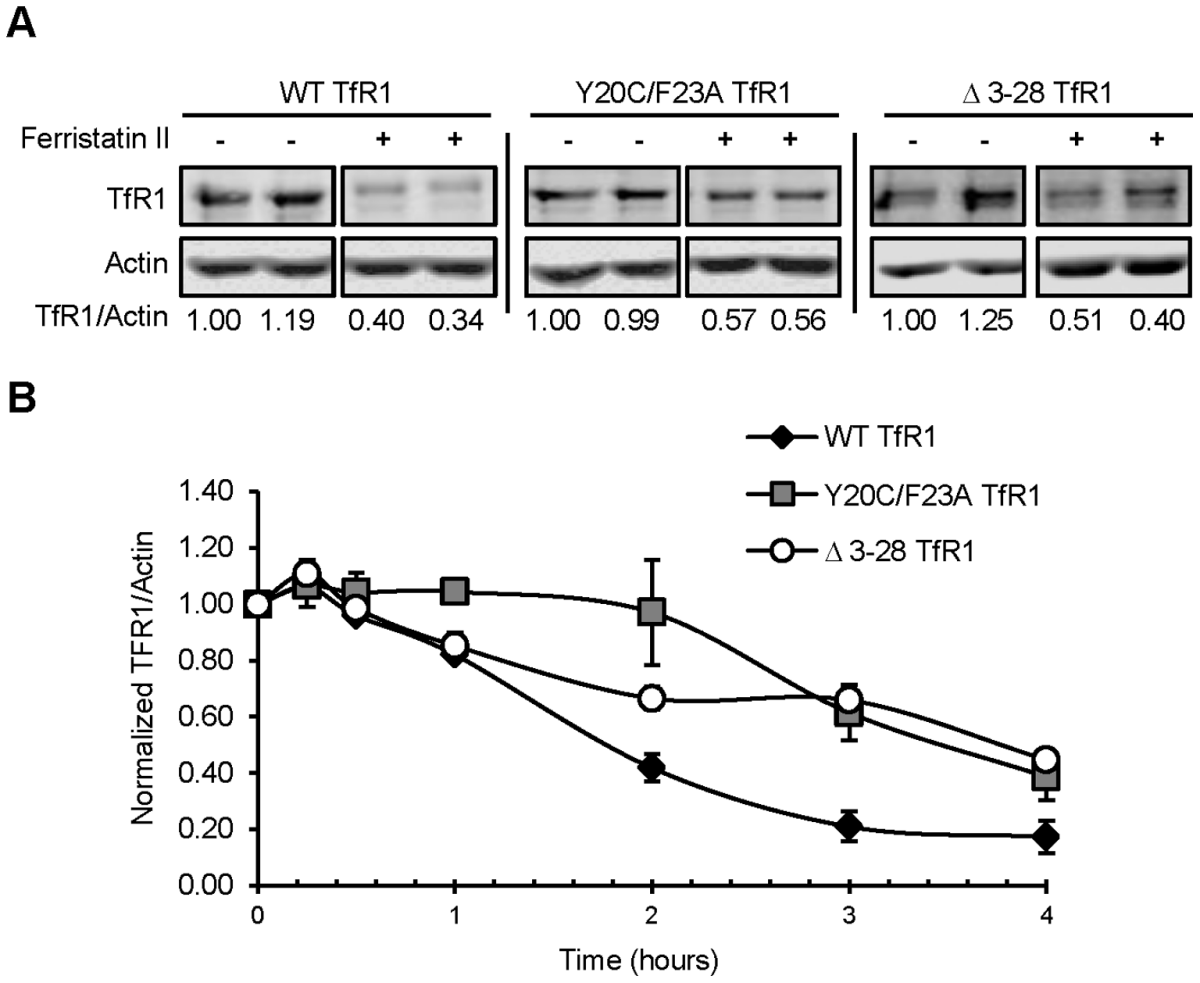
Ferristatin induced degradation is independent of an interaction with the clathrin endocytic machinery. Panel A: TRVb cells stably transfected to express WT TfR1, Y20C/F23A TfR1, or Δ3–28 TfR1 were treated for 4 hours with 50 µM ferristatin II. Density ratios for TfR1/Actin normalized to control lanes (DMSO treated) in the absence of ferristatin II are indicated for each lane. Nonconsecutive lanes are separated by a white space. Individual blots are separated by a black bar. All blots were probed using a sheep-TfR1 antibody raised against the ectodomain of TfR1. Panel B: Time course of WT TfR1, Y20C/F23A TfR1 and Δ3–28 TfR1 degradation after 0–4 hour incubation with 50 µM ferristatin II. Shown are mean TfR1/Actin ratios ± SEM from 3 separate experiments performed in duplicate.

Two “internalization defective” TfR1 mutants were studied. Y20C/F23A TfR1 harbors point mutations within the receptor’s endocytic motif, while Δ3–28 TfR1 is a deletion mutant lacking 25 amino acids of the domain [21]. This domain is responsible for interaction with AP-2 and the clathrin machinery. Previous investigations by McGraw and coworkers established stable expression of these mutants in TRVb cells, which lack endogenous Tf receptors [46]. Effects of ferristatin II on TRVb cells expressing the Y20C/F23A or Δ3–28 TfR1 mutants were compared to TRVb1 cells expressing wild-type TfR1 as a control (Figure 4A). Both receptor mutants were degraded upon ferristatin II treatment, although the time course of degradation was delayed compared to wild-type TfR1 (Figure 4B).

### Tf Blocks Ferristatin II Action

**Figure 5:**
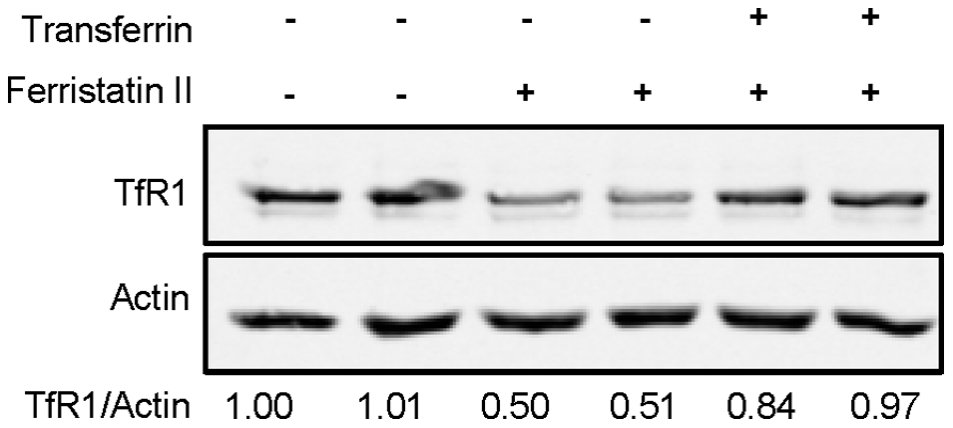
Tf blocks ferristatin II induced receptor degradation. HeLa cells were treated for 4 hours with 50 µM ferristatin II with or without 1 mg/mL Tf. Shown below are the density ratios for TfR1/Actin normalized to control lanes (DMSO treated) in the absence of ferristatin II. Shown is a representative blot with similar results observed on several separate occasions.

**Figure 6:**
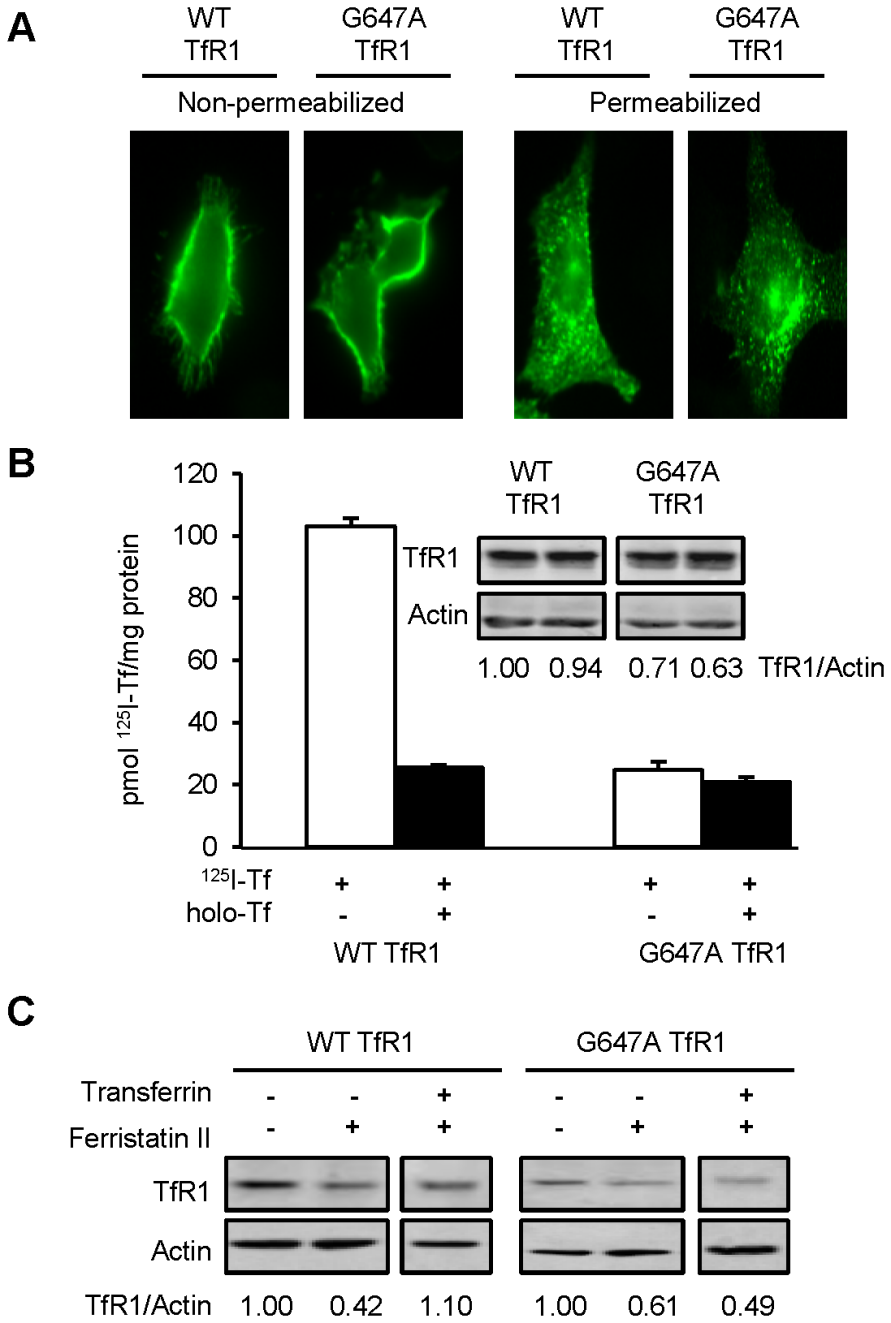
Ferristatin II induces degradation of ligand binding mutant G647A TfR1. Panel A: TRVb cells were transfected with 1 µg WT TfR1 or G647A TfR1, plated on poly-L-lysine coated cover slips and fixed with 4% paraformaldehyde. Cells were immunoreacted with OKT9 (α-TfR1) followed by goat anti-mouse Alexa 488 and imaged using a Zeiss Observer Z1 Axioscope microscope. Panel B: TRVb cells were transfected as in (A) in 6-well plates and incubated for 48 hours (see Experimental Procedures for details). Cells were then chilled, washed and incubated with 500 nM ^125^I-Tf in the absence or presence of 5 µM unlabeled Tf for 2 hours. After washing and lysis, cell associated radioactivity was measured by γ-counting. Absolute deviation for duplicate values for cells with 500 nM ^125^I-Tf (open bars) or 500 nM ^125^I-Tf +5 µM Tf (closed bars) is shown. Inset: Western blot confirms equivalent expression levels for WT and G647A TfR1. Panel C: TRVb cells, transfected with WT or G647A TfR1 were treated for 4 hours with 50 µM ferristatin II with or without 1 mg/mL Tf. Shown below are the density ratios for TfR1/Actin normalized to control lanes (DMSO treated) in the absence of ferristatin II. Nonconsecutive lanes are separated by a white space. Blot is representative of several experiments (n = 6, WT and G647A TfR1).

Since it is known that clathrin-mediated endocytosis of TfR1 occurs regardless of receptor occupancy [56], [57], [58], we examined the effects of Tf on TfR1 degradation by ferristatin II. In the presence of holo-Tf, the action of ferristatin II to induce degradation of TfR1 was blocked (Figure 5). There are at least two possible explanations for the observed antagonism: ferristatin II competes with holo-Tf for binding to TfR1 or Tf acts as a non-specific antagonist of ferristatin II action. Therefore, a receptor point mutant, G647A TfR1, was constructed to probe the receptor’s ligand-binding domain. Gly647 resides in the RGD sequence previously shown to be critical for Tf binding [59], [60]. The ability of ferristatin II to degrade wild type and G647A TfR1 was tested in TRVb cells. Transiently expressed G647A TfR1 was distributed in these cells in a fashion similar to wild type as shown by immunofluorescence microscopy (Figure 6A). As predicted, the mutant receptor failed to bind ^125^I-labeled Tf (Figure 6B). Cells transiently transfected to express either wild-type receptor or the G647A mutant were treated with or without 50 µM ferristatin II in the presence or absence of holo-Tf. Western blot analysis confirmed that holo-Tf blocked ferristatin II induced degradation of wild-type TfR1 but it did not interfere with G647A TfR1 degradation (Figure 6C). Therefore, binding of ligand to the receptor blocks ferristatin II action.

### Effects of Ferristatin II *in vivo*

**Figure 7:**
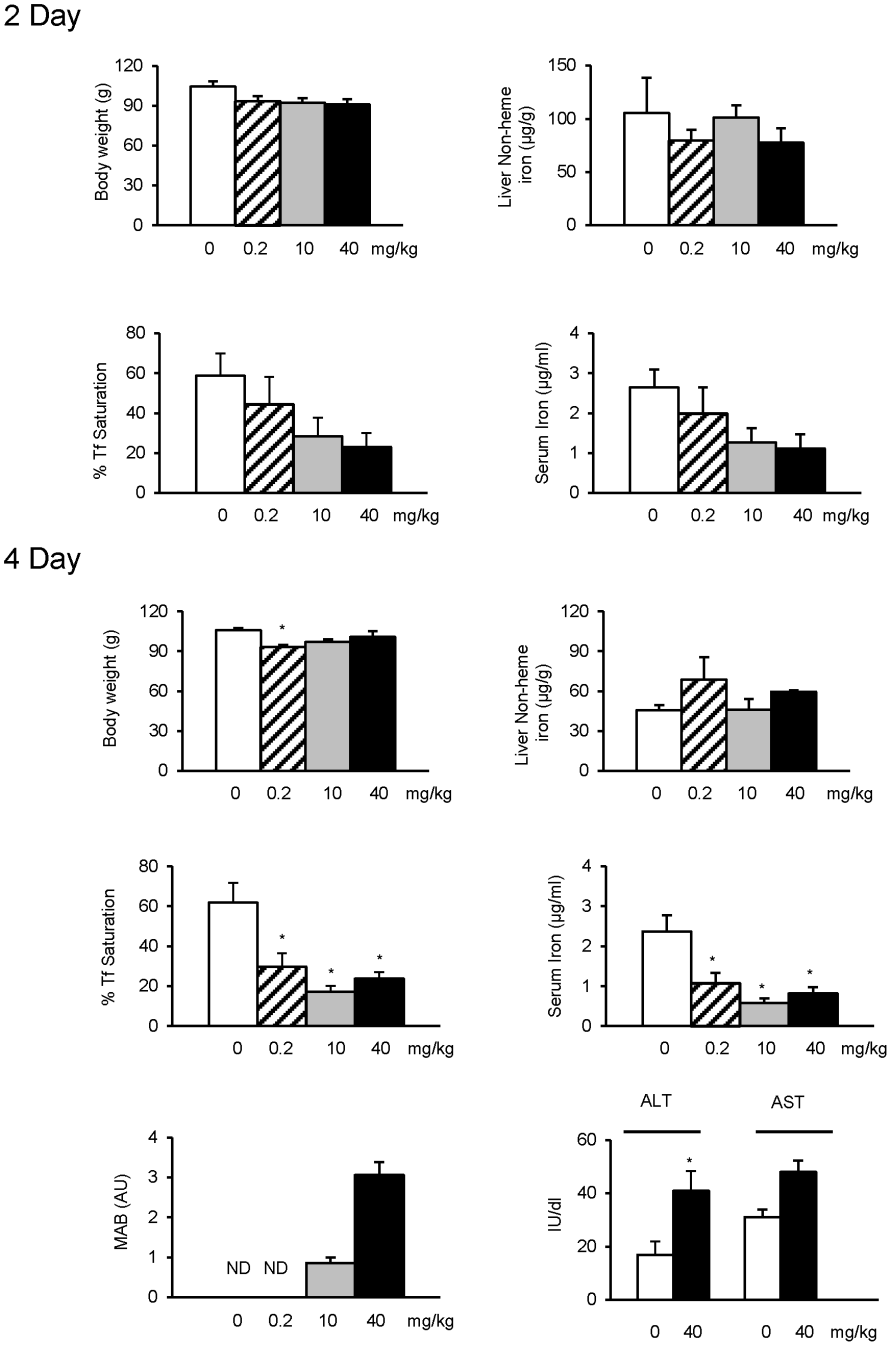
Effects of ferristatin ll *in vivo*. Rats were treated with vehicle control (saline) or 0.2, 10 or 40 mg/kg ferristatin ll for 2 or 4 days as described in Experimental Procedures. Shown are mean values ± SEM (n = 4) for body weight (*P = 0.022), liver non-heme iron, Tf saturation (*P = 0.018, 0.002 and 0.006 for 0.2, 10 and 40 mg/kg, respectively) and serum iron levels (*P = 0.016, 0.002 and 0.005 for 0.2, 10 and 40 mg/kg, respectively) measured after a 6 h fasting period. P values were determined by one-way ANOVA followed by Tukey’s test as a post hoc comparison. Monoacetylbenzidine (MAB) levels in rat urine were determined by HPLC and normalized to creatinine levels (arbitrary units). Shown are means ± SEM (AU = arbitrary units; ND = not detected. Serum ALT and AST activities were determined using ALT and AST reagents (Thermo Scientific) for rats injected for 4 days with vehicle or 40 mg/kg ferristatin II. Shown are means ± SEM (n = 3–6; *P = 0.033 determined by two-tailed Student’s t test).

To examine whether effects of ferristatin II observed *in vitro* reflect its activity *in vivo*, rats were injected twice daily with vehicle, 0.2, 10 and 40 mg/kg of the drug for one or three days, then once in the morning of the second or fourth day prior to tissue collection. Monoacetylbenzidine, a known metabolite [61], was detected in urine after 4 days of treatment at higher doses. Serum ALT and AST activities were measured to assess possible liver damage; serum ALT was slightly elevated at the highest dose but AST was not significantly affected (Figure 7). These results demonstrate the drug is metabolized by acetylation with minimal toxicity over the time course and doses administered in our experiments.

**Figure 8:**
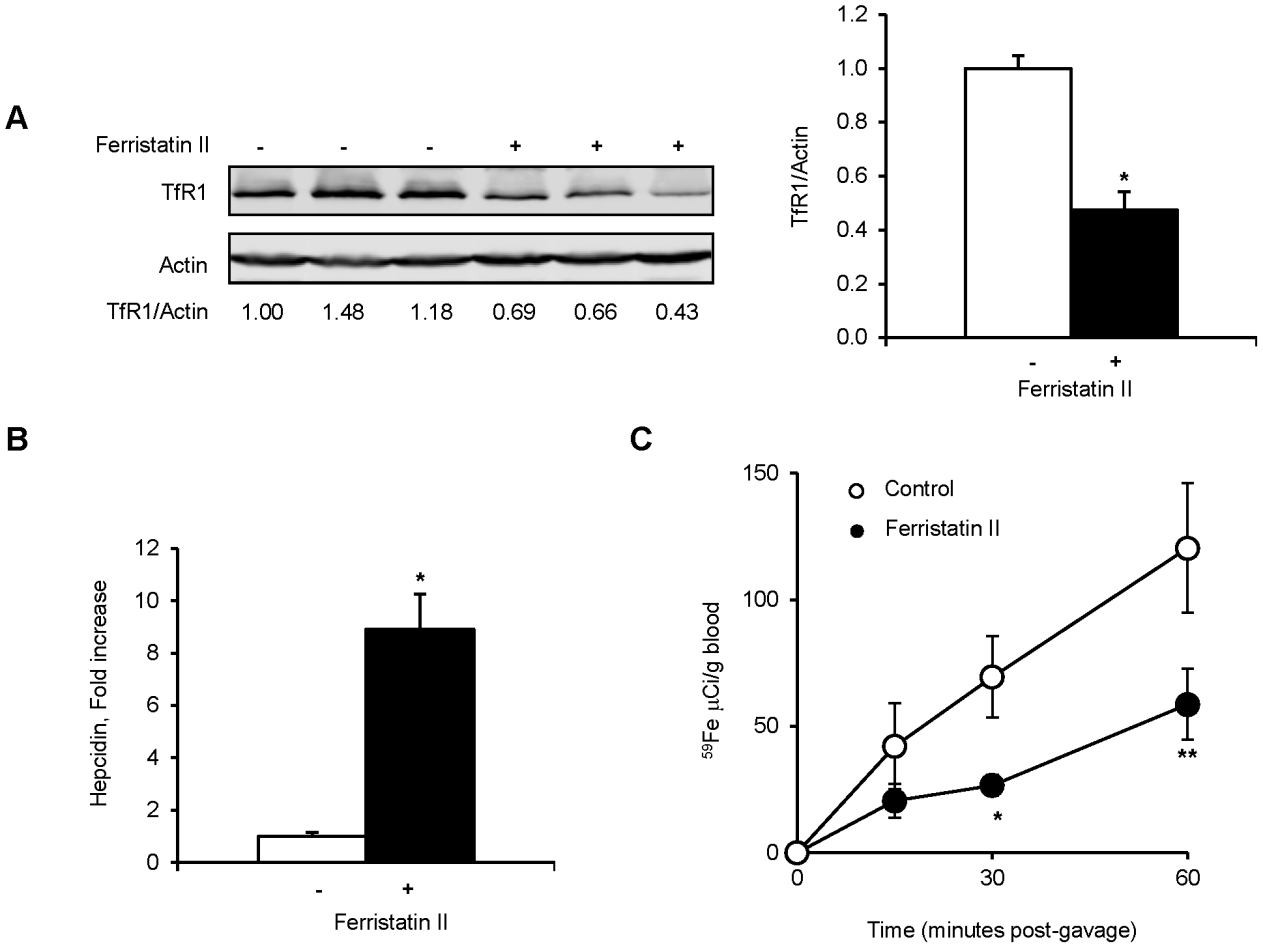
Ferristatin II alters iron homeostasis *in vivo*. Panel A: Liver lysates from rats injected with saline or 40 mg/kg ferristatin ll for 4 days were immunoblotted to determine TfR1 levels. Actin was used as a loading control. Bar graph shows normalized TfR1/actin ratio as the mean ± SEM (n = 7; *P<0.001 determined by two-tailed Student’s t test). Panel B: Liver RNA was isolated, reverse transcribed and qPCR performed as described under Experimental Procedures. Data were normalized to levels of 36B4. Shown are means ± SEM (n = 5–7; *P<0.001 determined by two-tailed Student’s t test). Panel C: To determine intestinal iron absorption, control and treated rats were fasted for 4 h and ^59^Fe was administered by gavage. Blood samples were drawn at indicated times and radioactivity was determined by gamma counting. Shown are means ± SEM for control (open circles; n = 4–6) and ferristatin ll (closed circles; n = 7–8; *P = 0.01 and **P = 0.05 determined by two-tailed Student’s t test).

Significant changes in liver non-heme iron levels were not observed over the course of ferristatin II treatment (Figure 7). However, Tf saturation and serum iron levels were reduced after 2 days of treatment and significantly lower at all concentrations tested for the 4 day treatments. Western blot analysis revealed TfR1 levels were significantly reduced in livers from treated rats compared to vehicle-injected controls, consistent with the action of ferristatin II to degrade receptors *in vitro* (Figure 8A). It has been proposed that dissociation of HFE from TfR1 promotes synthesis of the iron regulatory hormone hepcidin [31], [32]. Hepcidin then acts on the iron exporter ferroportin to reduce systemic iron levels. qPCR analysis revealed that hepcidin synthesis was enhanced in rats treated with ferristatin II with ∼ 9-fold increase in mRNA levels relative to control (vehicle-injected) rats (Figure 8B). Reduced intestinal iron absorption was also observed in rats treated with ferristatin II in uptake experiments that determined the amount of ^59^Fe in blood after intragastric gavage (Figure 8C).

## Discussion

It has been long established that TfR1 enters cells through clathrin-mediated endocytosis [6]. This pathway is particularly well understood in the context of cellular iron delivery [62]. However, under some circumstances cell surface proteins like epidermal growth factor receptor (EGFR) [63], the glucose transporter GLUT4 [64] and TGF-β family members [65] are internalized via lipid rafts in addition to clathrin dependent mechanisms. Early observations with the first iron transport inhibitor, ferristatin, indicated that like these other membrane proteins, TfR1 could undergo lipid raft mediated internalization [44]. Ferristatin induced degradation of TfR1 was sensitive to filipin and nystatin, two cholesterol-depleting drugs that implicate a role for lipid rafts. Although nystatin-sensitivity has been noted in some examples of clathrin-mediated endocytosis [66], knockdown of clathrin did not interfere with TfR1 degradation induced by ferristatin [44], further supporting the function of the lipid raft pathway in the drug’s action. Our characterization of ferristatin II activity reveals that this second member of the ferristatin family of iron transport inhibitors induces degradation of TfR1 both *in vivo* and *in vitro*. The loss of receptors explains why cells in culture treated with ferristatin II have lower ^55^Fe uptake from Tf. Our results further confirm sensitivity of the degradation pathway to nystatin. Our studies of TRVb1 cells stably expressing various receptor mutants defective in clathrin-mediated endocytosis [21] suggest that elements of the TfR1 cytoplasmic domain necessary for clathrin-mediated endocytosis may not be required for ferristatin II-induced degradation. These independent lines of evidence indicate that ferristatin II mediates degradation of TfR1 by a non-clathrin pathway, and a role for lipid rafts is suggested by the sensitivity to cholesterol depletion.

The importance of lipid rafts in iron metabolism and signaling is just beginning to be elucidated. For example, degradation of the iron exporter ferroportin induced by the iron regulatory hormone hepcidin is disrupted by cholesterol depletion [67] and ferroportin has been shown to fractionate in detergent-resistant membrane fractions with flotillin or caveolin-1, both markers of lipid rafts [67]. Finally, the cholesterol binding protein CD133 is known to influence Tf uptake in Caco-2 cells [68]. Such evidence suggests a prominent role for lipid rafts in the regulation of iron metabolism. Regulation of the lipid raft pathway by iron status provides an additional layer in the complex regulation of iron transport. The ability to pharmacologically induce an alternative TfR1 membrane trafficking mechanism through lipid rafts provides an interesting new avenue to modulate the delivery of iron with potential clinical application.

Unlike clathrin-mediated TfR1 endocytosis, which occurs independent of receptor occupancy, the presence of Tf antagonizes ferristatin II-induced degradation. The finding that G647A TfR1 with defective ligand binding is susceptible to ferristatin II suggests that Tf may occlude the drug’s interactions with wild-type TfR1. Alternatively, Tf interactions with the receptor could induce conformational changes that block ferristatin II binding to a distal domain, possibly including the cytoplasmic domain. Further work is necessary to more precisely define structural elements of TfR1 that contribute to targeting by ferristatin II. Our results indicate these effects are quite specific since ferristatin II does not affect protein levels of TfR2 expressed in Hep3B cells. Although structurally related, TfR1 and TfR2 differ in domain interactions with both HFE and Tf [47]. Sensitivity to degradation by ferristatin II marks another structural distinction between the two receptors that should be further explored.

**Figure 9:**
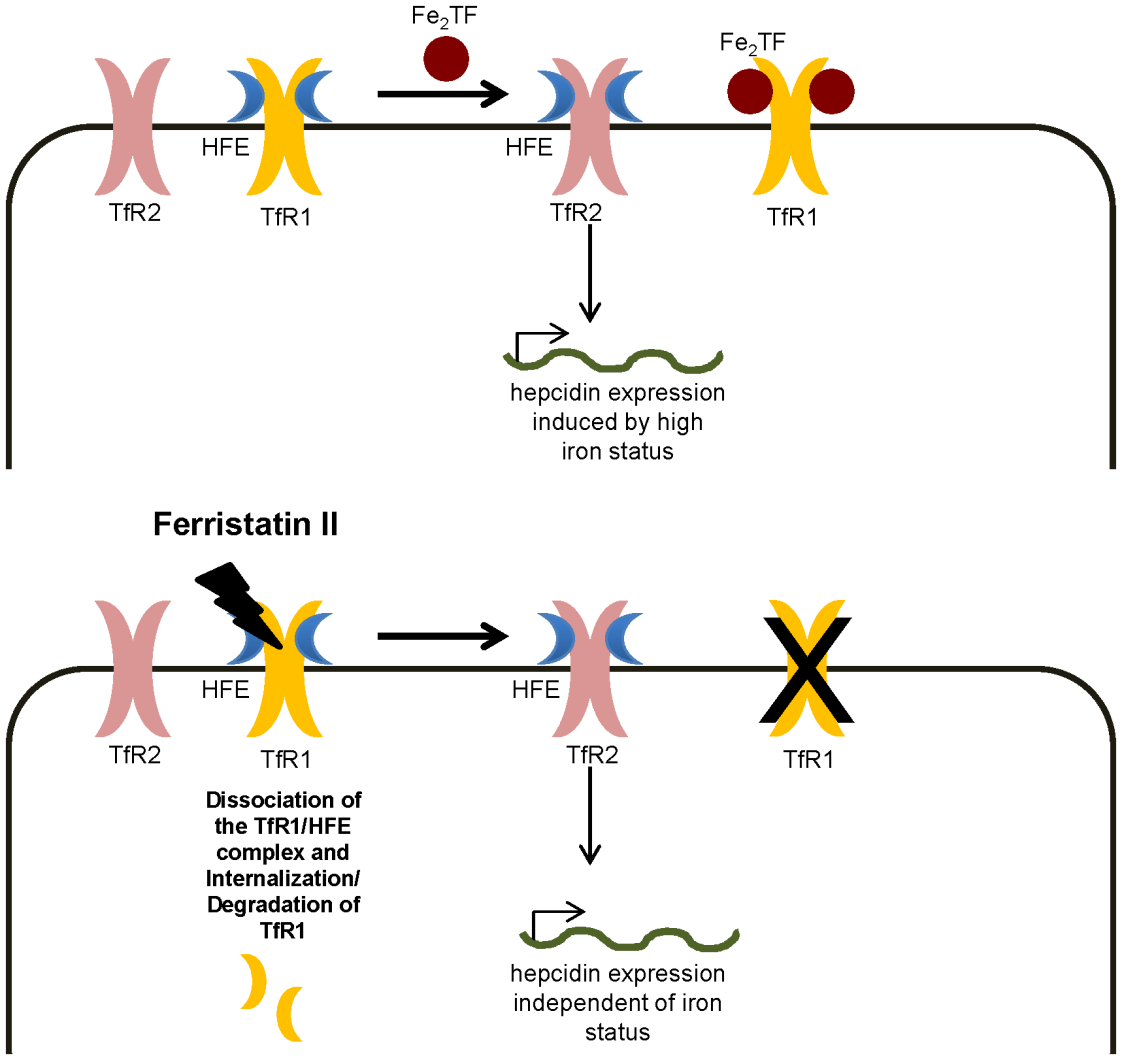
Model of iron homeostasis under normal and ferristatin II conditions. The contributors to iron metabolism include TfR1, TfR2, Tf and HFE. These molecules work together to maintain systemic iron homeostasis by sensing high and low iron levels. See text for details.

The ability of ferristatin II to reduce TfR1 levels *in vivo* was confirmed by administering the drug to rats at doses up to 40 mg/kg. Serum iron and transferrin saturation were lowered after 2 days of treatment, and significantly reduced at all concentrations of ferristatin II tested after 4 days of treatment. These effects were associated with a ∼50% decrease in the protein level of TfR1 in liver. Notably, hepatic non-heme iron content did not change. The lack of change in hepatic iron despite hypoferremia is consistent with a block in iron mobilization. Our intragastric gavage experiments show that rats treated with ferristatin II also have reduced intestinal uptake of ^59^Fe to the blood. Both of these observations can be explained by the observed up-regulation of hepcidin, a regulatory hormone of iron metabolism [38]. Hepcidin interacts with the iron exporter ferroportin to induce its lysosomal degradation, thereby blocking dietary absorption [35] and release of iron stores [69]. Hepcidin is known to be induced by increasing Tf saturation under high iron conditions which triggers dissociation of HFE from TfR1 [31] coupled to its association with stabilized TfR2 [32]. Since ferristatin II degrades TfR1 but not HFE, we hypothesize the action of ferristatin II releases HFE to promote hepcidin synthesis. In this scenario, receptor degradation rather than high iron promotes HFE association with TfR2, and possibly activates other factors that regulate hepcidin expression [70], [71]. As shown in Figure 9, under basal conditions, HFE is bound to TfR1. As levels of iron saturated Tf increase, Tf out-competes HFE for binding to TfR1. Released HFE can bind TfR2 to initiate a signaling cascade promoting hepcidin synthesis. In the presence of ferristatin II, the degradation of TfR1 liberates HFE to bind TfR2 and induce hepcidin synthesis in the liver. This action appears to be independent of high iron, since rats treated with ferristatin II display hypoferremia yet continue to upregulate hepcidin synthesis.

It is important to consider that other targets of ferristatin II action may contribute to the effects we observe. *In vitro* studies have shown that ferristatin II also inhibits transport of iron by DMT1 [45], and this transporter plays an important role in apical iron uptake by enterocytes [72], [73]. Inhibition of DMT1 activity would reduce iron absorption, leading to lower serum iron and transferrin saturation independent of hepcidin’s action on ferroportin. On the other hand, some reports have suggested that hepcidin also regulates DMT1 function in the intestine [74], so that direct suppression of DMT1 might also arise due to hepcidin induction by ferristatin II. Regardless of the precise target(s), inhibition of import and/or export of iron across the intestinal mucosa provides a rational explanation for the reduced level of circulating iron observed in rats treated with ferristatin II. The idea that increased hepcidin blocks iron mobilization from stores, a known action of the hormone (67), is also consistent with the observation that non-heme iron levels in liver are not altered by ferristatin II treatment.

Several other pharmacological tools now have been developed to target the hepcidin axis [75], including “mini-hepcidins” that down-regulate ferroportin [76], [77], BMP inhibitors that block Smad signaling [78], [79], and agents that perturb Stat signaling [80], [81]. The ferristatins are unique in targeting TfR1 degradation [43], [44]. We previously have shown that degradation of the receptor induced by ferristatin occurs through a lipid raft-dependent mechanism [44]. Recently, the antimalarial agent dihydroartemisinin was shown to degrade TfR1 [82]. Depletion of cellular iron through this mechanism was proposed to account for this drug’s anticancer effects. Interestingly, down-regulation of TfR1 by dihydroartemisinin also was shown to proceed via a lipid raft mechanism. The alternative TfR1 membrane trafficking mechanism revealed by use of small molecule inhibitors provides an intriguing new pathway to potentially regulate iron metabolism in a number of different disease states.
